# Hybrid quasi-3D optimization of grid architecture for single junction photovoltaic converters

**DOI:** 10.1007/s11082-021-02850-x

**Published:** 2021-04-12

**Authors:** Veikka Nikander, Jianguo Wei, Arto Aho, Ville Polojarvi, Antti Tukiainen, Mircea Guina

**Affiliations:** 1 Optoelectronics Research Centre (ORC), Physics Unit, Tampere University, Korkeakoulunkatu 3, 33720 Tampere, Finland; 2 Global Energy Interconnection Research Institute Europe GmbH, Kantstr. 162, 10623 Berlin, Germany

**Keywords:** Photovoltaic converter, Quasi-3D simulation, Front contact grid optimization, Solcore, Super Gaussian illumination, Power over fiber

## Abstract

A numerical study of metal front contacts grid spacing for photovoltaic (PV) converter of relatively small area is presented. The model is constructed based on Solcore, an open-source Python-based library. A three-step-process is developed to create a hybrid quasi-3D model. The grid spacing under various operating conditions was assessed for two similar p–n and n–p structures. The key target was finding optimal configuration to achieve the highest conversion efficiency at different temperatures and illumination profiles. The results show that the n–p structure yields wider optimal spacing range and the highest output power. Also, it was found that temperature increase and illumination nonuniformity results in narrower optimal spacing for both structure architectures. Analyzing the current–voltage characteristics, reveals that resistive losses are the dominant loss mechanism bringing restriction in terms of ability to handle nonuniform illumination.

## Introduction

Photovoltaic (PV) converters are devices based on semiconductor p–n junctions, operating on the similar principle as the solar cells, yet with the main difference in terms of illumination, i.e. PV converters being illuminated by a light beam with a narrow spectrum, which is typically delivered by a laser source (Beattie et al. 2021; DeLoach et al. 1978; Helmers et al. 2020; Oliva et al. 2008; Zhao et al. 2016). The main application for PV converters is remote power delivery. The electrical power is first converted into laser light, which is then transferred through an optical fiber or free space to the PV converter that transforms the light back to electricity. Power beaming systems have an advantage over traditional electric power transfer for example in applications that require galvanic isolation (De Nazaré and Werneck 2012), low electromagnetic interference, or sparkless operation.

The optimal PV converter design relies on one hand on the optimization of the layer structure, being similar in many aspects (i.e. current density, size etc.) to the guidelines used when designing solar cells for concentrated sunlight operation. In such applications, the design of the front metal grid, which should generally ensure efficient charge collection with minimal voltage loss, is an essential optimization parameter. Grid design becomes even more crucial for a PV converter given the high light excitation intensity, relatively small device area, and geometry matching the beam shape delivered by the fiber optics elements.

**Fig. 1:**
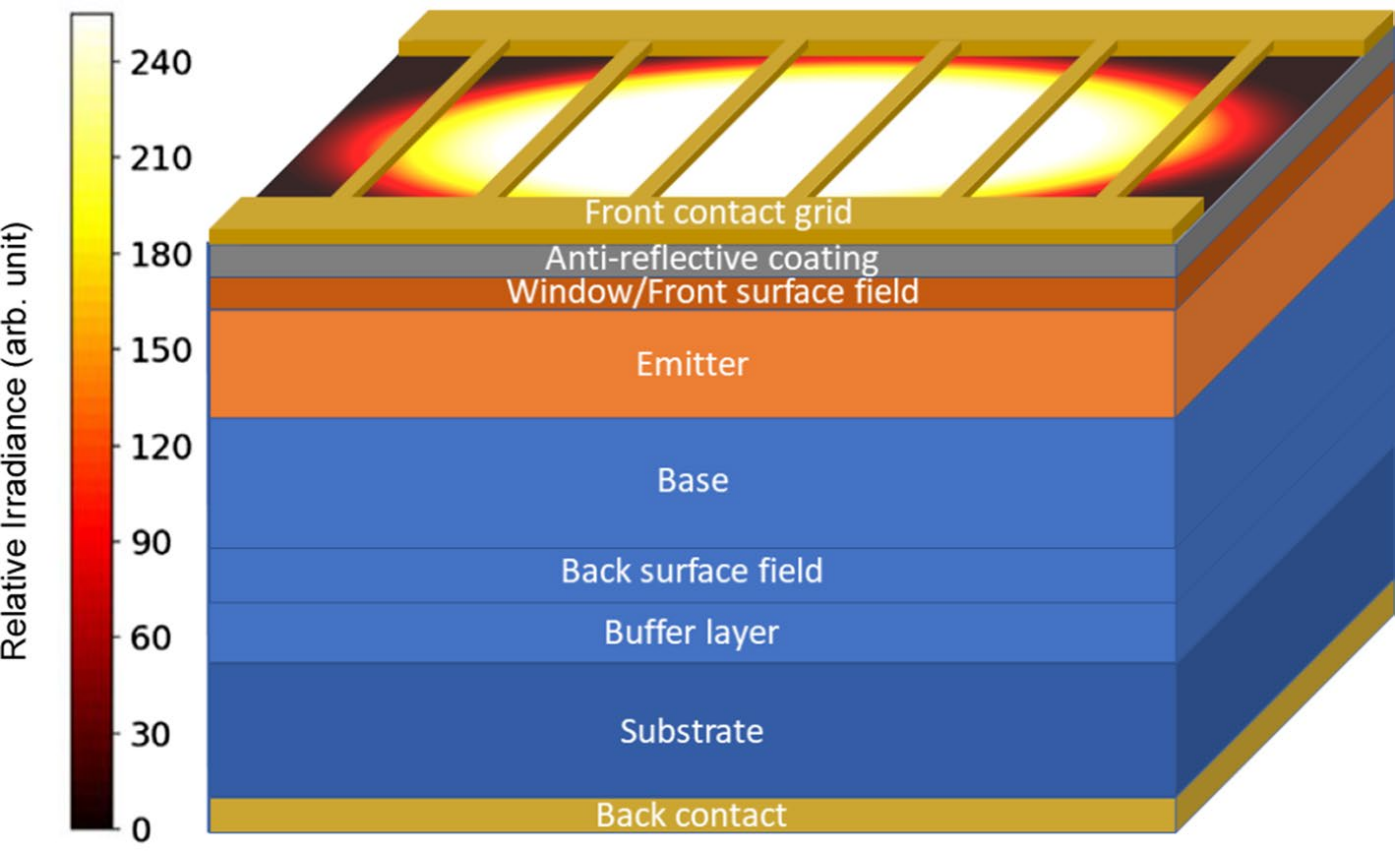
A sketch of the HQ3D models input parameters consisting of semiconductor layer structure, front con-tact metal grid profile and illumination irradiance profile

Here, a numerical study of front contact grid spacing for gallium arsenide (GaAs) based PV converters employing a hybrid quasi-3D (HQ3D) model is presented. The model is based on Solcore (Alonso-Álvarez et al. 2018) and a double diode model (DDM) fitting. Solcore is an open-source Python-based library for PV modeling with multiple simulation tools including, e.g., Transfer Matrix Method (TMM) (Byrnes 2016), Fortran-based Poisson–Drift–Diffusion (PDD) solver (Gartland 1993), and SPICE-based Quasi-3D solver. All models from Solcore were used with default settings if not otherwise stated. Solcore version v5.6.0 was used in the present work. The HQ3D model calculates from the given device structure, grid profile, and illumination profile the current–voltage (*I*–*V*) characteristics for the cell and a detailed 2D voltage map over the surface of the device. An example of input parameters is given in Fig. 1. From the simulation results, it is possible to detect the areas of higher losses and most importantly get the efficiency of the simulated device. Optimization is done for similar p–n and n–p polarized structures with different temperatures and illumination profiles. It is important to understand the temperature dependence of the device characteristics as the required cooling, working environment, and illumination profile vary depending on the application where the converter is used. For example, the output from an optical fiber shows often super-Gaussian distribution (Daido et al. 1979; Fujii et al. 1984; Gloge and Marcatili 1973) and with free space applications, one might favor normal Gaussian distribution due to its smaller divergence (Parent et al. 1992). One might also need to have a uniform illumination profile when the cell is rectangular or a part of a cell–matrix.

## Hybrid quasi-3D model

The HQ3D modeling starts by determining the devices 1D characteristics using TMM and PDD solvers. TMM is used for calculating reflection, absorption and transmission of the incident light. The absorption, i.e., carrier generation profile and structural elements with the appropriate material parameters are then passed to the PDD solver which calculates the internal quantum efficiency and *I*–*V* characteristics of the 1D structure. After the 1D behavior of the device is determined, a DDM is constructed from the PDD-created *I*–*V* characteristics by fitting a double-diode equation to it using Eq. (1),

1 $$I = I_{L} - I_{01} e^{{\frac{{q\left( {V + IR_{s} } \right)}}{kT}}} - I_{02} e^{{\frac{{q\left( {V + IR_{s} } \right)}}{2kT}}} - \frac{{V + IR_{s} }}{{R_{sh} }},$$

where ${I}_{L}$ is light generated current, ${I}_{01}$ and ${I}_{02}$ are reverse saturation currents for diode 1 and 2, respectively, ${R}_{s}$ is the series resistance of the device, ${R}_{sh}$ is the shunt resistance,$T$ is temperature of the cell, $q$ is elementary charge and $k$ is the Boltzmann constant. Fitting is done by using a highly efficient method (Gao et al. 2016) utilizing Lambert *W*-function (Corless et al. 1996) and Nelder–Mead method (Nelder and Mead 1965).

After the DDM is constructed, a circuit equivalent quasi-3D structure is determined. This structure models the flow of current inside the device and takes also into account the effects of the front metal grid and illumination profile. The cell is divided in the middle of the finger spacing to parts consisting of one finger each. This division is done for faster calculation time. Sub cells are then inserted into the Quasi-3D solver which discretizes the area to smaller sub-devices in the growth plane. The sub-devices are connected to each other by resistors that model the lateral current flow. Resistance values of the connecting resistors are determined using the sheet resistance formula

2 $$\rho_{sh} = \frac{1}{qd\mu N},$$

where $q$ is charge, $d$ is thickness, $\mu$ majority carrier mobility and $N$ doping of given layer. Active doping levels of the layers are used in the determination of the sheet resistance. Sheet resistance values for layers above the junction are key parameters in the grid optimization since they determine the resistive losses in lateral current transport.

Illumination and grid profile for each device is determined based on specific profile matrices. These matrices can be created mathematically or extracted from experimental data converted to greyscale images. The illumination matrix scales the light-generated current calculated with PDD to subparts. The grid profile matrix determines the place of contact fingers and the busbar. Parts of the device that are underneath the contact grid have no light-generated current. The final circuit equivalent model is then given to a SPICE solver, ngspice (*Ngspice)*, which calculates the total *I*–*V* characteristics of the device and voltage map over the surface of the device and metal contacts.

## Optimization case study

### Grid optimization

Front contact grid metallization is an important part of PV cell design ensuring minimal electric losses when collecting the carriers to external circuit trough conductive material, such as gold or silver. On the other hand, the metallization gives results to an important loss mechanism through shading the PV converter, as metals are not transparent to light for most of the solar spectrum. Even though there is a lot of progress done in the development of transparent conductive materials (Kohler et al. 2020; Morales-Masis et al. 2015; Saive et al. 2016), using metals represents the most common method to realize the contact grid. Yet, the trade-off between ensuring low resistivity and minimal shadowing can be optimized through geometry of the grid contact for specific operation condition (i.e. spectrum, power density, temperature). In this work, we have considered a double busbar finger grid pattern seen in Fig. 1 due to its simplicity.

For a traditional two-busbar finger architecture, the finger spacing is the key parameter to extract from the simulation. Optimal finger spacing can be extracted by constructing a 2D spacing–output power plot by running HQ3D simulations with different values of finger spacing. From the simulations, we then can extract the optimal spacing for example by using simple polynomial fit interpolation for the results.

### Simulated structures

**Fig. 2:**
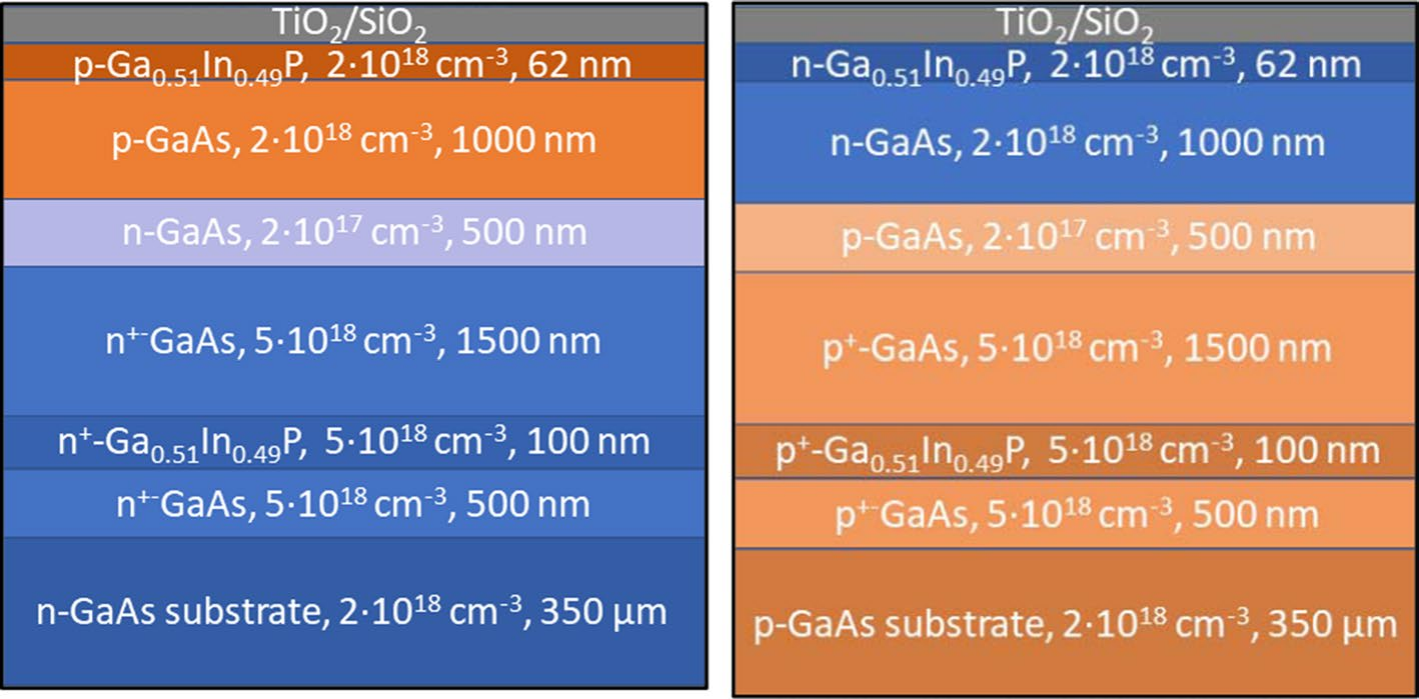
Simulated p–n and n–p structures

The optimization case study was done for a p–n (Vänttinen et al., 1995) and an otherwise similar but inverted n–p polarized PV converter structures. The layer structures of the studied devices are shown in Fig. 2. Simulated cell has area of 25 mm^2^ and has gold as finger metal with finger cross-section of 4.5 µm^2^. Structure was discretized to consist of 500 pixels parallel to the fingers and 1667 pixels perpendicular to them. Only a quarter of the cell was simulated for enhanced calculation speed by utilizing the contact grid’s symmetry.

Resistive losses from the busbars and other contacts are neglected in this study. Solcore’s material database was used in the simulations except for gold’s conductivity which was from (Raymond 1998). The band parameters were obtained from (Vurgaftman et al. 2001), the mobility parameters from (Sotoodeh et al. 2000), and refractive index data from (Software Spectra Inc. 2008).

### Illumination profile

**Fig. 3:**
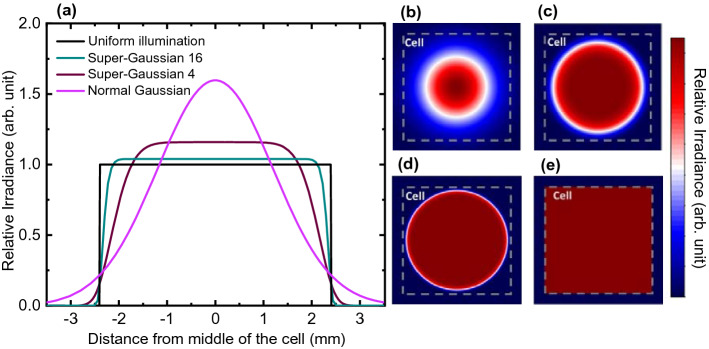
**a** Cross-sections of illumination profiles used in the optimization case study. **b** Normal Gaussian, **c** Gaussian order 4, **d** Gaussian order 16 and **e** uniform illumination profiles

We have considered super-Gaussian distributed illumination profiles, common for output profiles of optical fibers. Its irradiance follows the distribution function

3 $$I\left( {r,z} \right) = I_{o} \left( {\frac{{w_{0} }}{w\left( z \right)}exp\left( { - \left( {\frac{{r^{2} }}{{w\left( z \right)^{2} }}} \right)^{g} } \right)} \right)^{2}$$

where

4 $$w\left( z \right) = w_{0} \sqrt {1 + \left( {\frac{z}{{z_{R} }}} \right)^{2} }$$

with

5 $$z_{R} = \frac{{\pi w_{0}^{2} n}}{\lambda }$$

given as function of the beam waist, ${w}_{0}$ at the focus point, $\lambda$ is the wavelength of the light, *r* is the distance from the center of the beam, *n* is the index of refraction of the travelling medium, $z$ is distance from the focus point and *g* is Gaussian order of the beam (Shealy and Hoffnagle 2006; Svelto 2010). In this work, illumination profiles with Gaussian orders of 1 (normal Gaussian), 4, and 16 are considered. Fully uniform illumination is also considered for comparison to an ideal illumination situation. Illumination profiles and their cross-sections are plotted in Fig. 3.

## Results and discussion

### Illumination profile effects

**Fig. 4:**
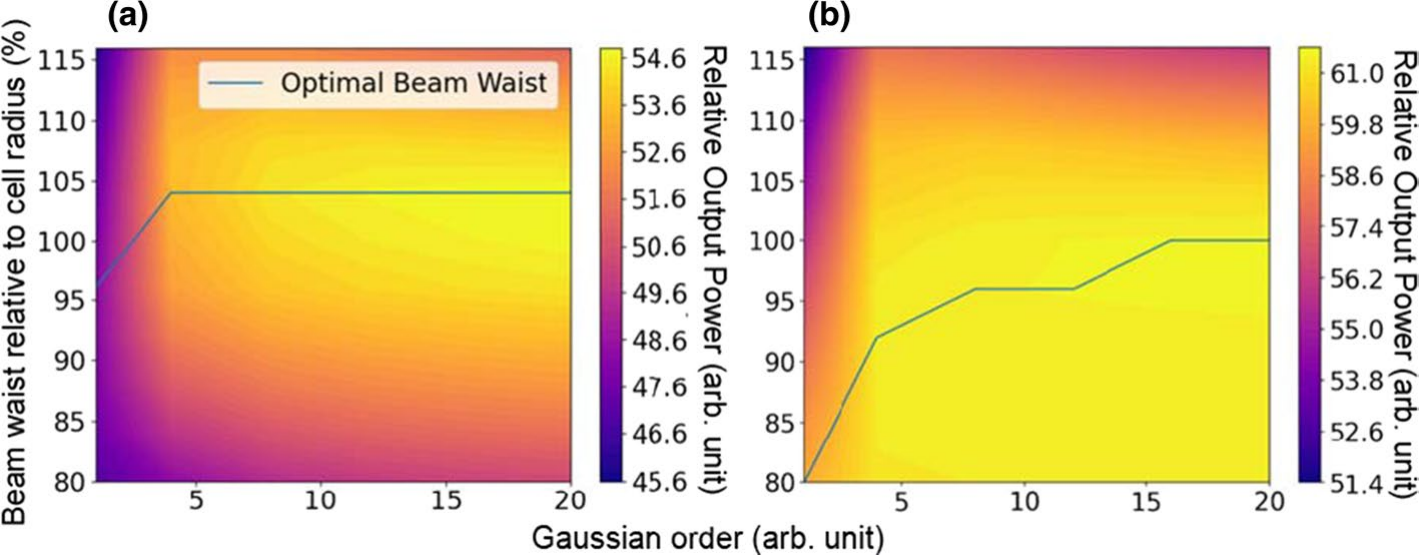
Relative output power map as function of beam waist relative to cell radius and Gaussian order **a** for p–n polarized structure and **b** for n–p structure. Line represents the optimal beam waist for given Gaussian order

Figure 4 shows the relative output power maps as a function of varying Gaussian order and beam waist for above mentioned structures calculated by the HQ3D model under 808 nm laser light with an average irradiance of 40 W/cm^2^ and at 300 K. Results are for illumination profiles at the focus point of Gaussian beam meaning $z=0$.

A significant difference was found between p–n and n–p structures. For the n–p structure HQ3D model predicts that the size of the beam waist does not matter much after it is of the similar size as the converter cell. With the p–n structure output power starts to notably decrease as the beam waist gets smaller. The optimal beam waist for the p–n structure is considerably higher than for n–p structure, being 104% for the Gaussian order 4 and higher. This indicates that the simulated p–n structure prefers uniform illumination even in the case of losing some power outside the cell edges.

### Grid optimization results

**Fig. 5:**
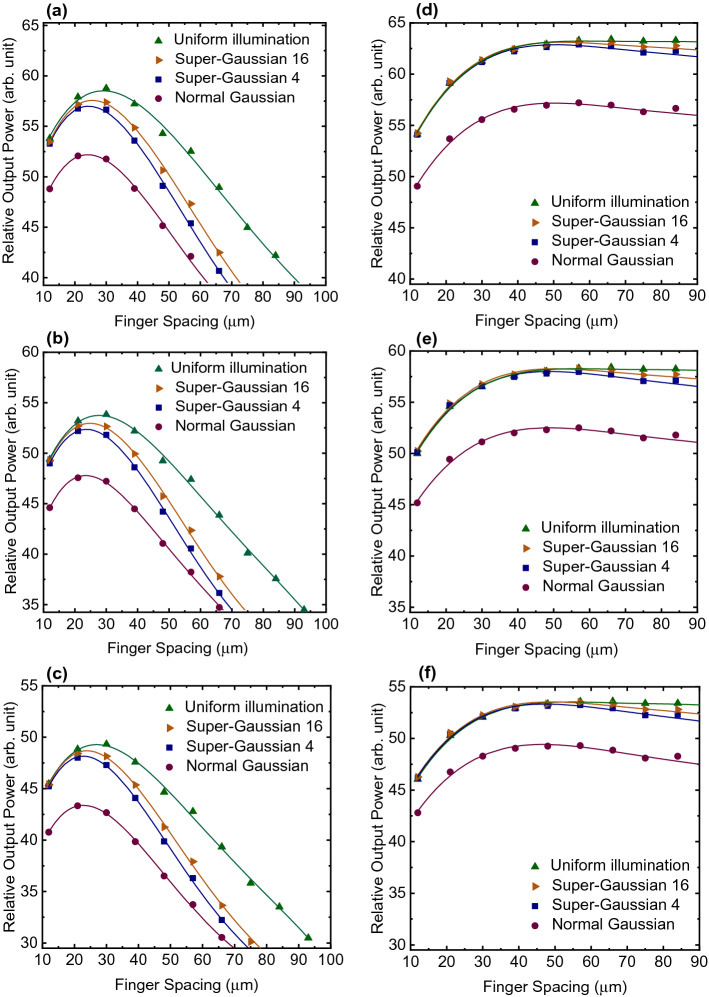
Grid optimization results calculated with HQ3D model (data points) and polynomial fit made for the data (lines) for p–n structure at **a** 300 K, **b** 325 K and **c** 350 K and for n–p structure at **d** 300 K, **e** 325 K and **f** 350 K

The simulations consider the same illumination with 808 nm laser light and an average irradiance of 40 W/cm^2^. A beam waist of 96% of the device radius was chosen based on output power maps. The same beam waist of 96% is used across all structures for easy comparison, although slightly higher output power would be expected for p–n structures with 104% beam waist size. The used waist size represents a compromise between optimal waist values for different illumination profiles. We considered three operation temperatures: ~ 300 K, ~ 325 K, and ~ 350 K. First temperature is chosen for easy comparisons with standard test conditions, second is a realistic target for achievable temperature for a passively cooled device and the third is a more pessimistic operating temperature for the converter. All these temperatures are possible operating temperatures for practical implementations depending on a cooling and an ambient temperature of the operating environment. Four different illumination profiles were used: normal Gaussian, Gaussian order 4, Gaussian order 16, and uniform distribution. This was done to yield a wide range of outcomes at different operating environments. A polynomial fit is employed for the simulated data points to extract more accurately the optimal spacings. Spacing-output power plots for all combinations of given situations are shown in Fig. 5. Optimal spacings extracted from the graph by polynomial fit and their corresponding maximum output powers are listed in Tables 1, 2, 3 and 4.

**Table 1:** Optimal spacings for p–n structure

Temperature (K)	Normal Gaussian (µm)	Super-Gaussian 4 (µm)	Super-Gaussian 16 (µm)	Uniform (µm)
300	24	25	26	29
325	24	24	25	28
350	23	23	24	27

**Table 2:** Optimal spacings for n–p structure

Temperature (K)	Normal Gaussian (µm)	Super-Gaussian 4 (µm)	Super-Gaussian 16 (µm)	Uniform (µm)
300	50	50	52	58
325	49	49	50	56
350	47	47	49	54

**Table 3:** Maximum power from p–n structure (arb. unit)

Temperature (K)	Normal Gaussian	Super-Gaussian 4	Super-Gaussian 16	Uniform
300	52.2	57.0	57.6	58.5
325	47.7	52.4	53.0	53.8
350	43.4	48.2	48.7	49.3

**Table 4:** Maximum power from n–p structure (arb. unit)

Temperature (K)	Normal Gaussian	Super-Gaussian 4	Super-Gaussian 16	Uniform
300	57.2	62.9	63.1	63.2
325	52.5	58.0	58.3	58.3
350	49.4	53.3	53.5	53.5

Both structures show decreased output power as finger spacing narrowed down. This power decrease originates from the shading effect of the fingers. But when the spacing is made wider, significant differences are seen between the p–n and n–p structures. As the spacing in p–n structure is made wider than 30 µm the output power starts to decrease dramatically but for the n–p structure output power remains relatively the same.

**Fig. 6:**
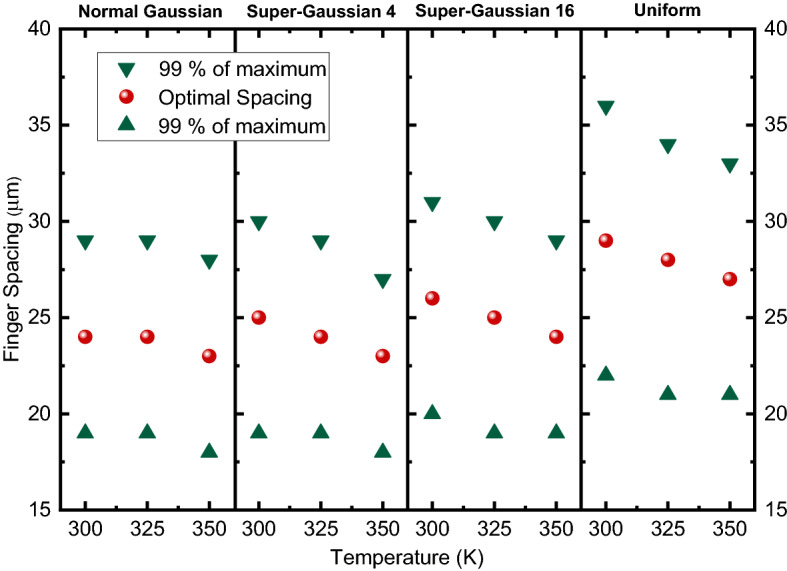
Optimal spacings as function of temperature for p–n structure

**Fig. 7:**
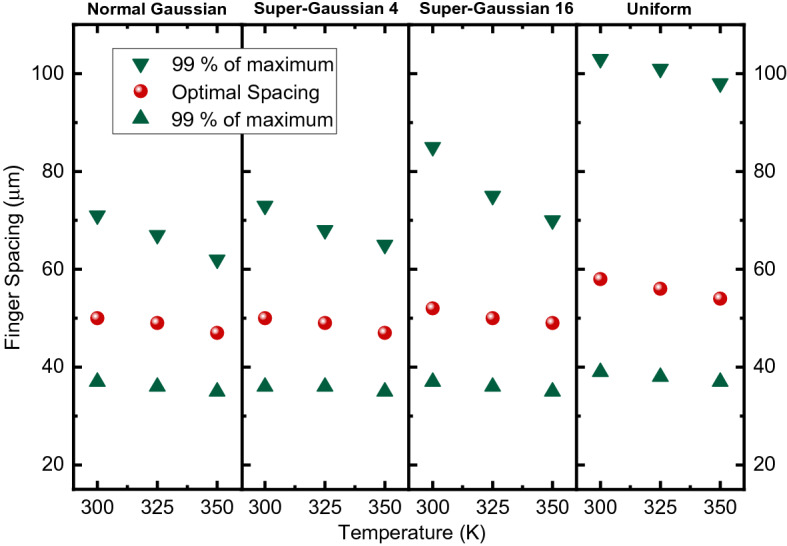
Optimal spacings as function of temperature for n–p structure

The n–p structure yields also considerably higher output powers giving the n–p structure about 10% power increase for a given structure, when compared to p–n structures. Temperature variation has also a significant effect on the output power of both structures. This is an expected result since PV cells have a significant drop in power as operating temperature increases (Luque and Hegedus 2010). Optimal spacings and margin for getting 99% of the maximum power as a function of temperature are shown in Figs. 6 and 7.

The simulations indicate that the optimal finger spacing decreases slightly as temperature increases. Also, it is found that the tolerance of spacings margin for getting 99% of maximum power decreases at higher temperatures.

Regarding the illumination profiles, the higher the beam uniformity the wider finger spacings can be used. This also leads to larger spacing tolerances and is thought to be caused by the fact that as illumination is more concentrated in the middle of the cell, the current passing through the contact fingers in the middle area of the cell increase compared to the edge areas. This leads to higher resistive losses in the fingers in the middle area, which needs to be compensated by adding more contact fingers and thus decreasing the finger spacing.

Also for the p–n structure, an improved uniformity of the illumination profile clearly yields higher output power but for the n–p structure there is no significant difference up to the widest spacing. One exception here is the normal Gaussian distribution which yields considerably less output power for both p–n and n–p structures. This is mainly due to large fraction of the excitation power that remains outside of the cell area. With the beam waist of 96% of cell radius, the normal Gaussian illumination profile leaks 7.3% of light outside the cell edges. This is far more than when super Gaussian of order 4, which leaks only 0.1%, is used.

### Analysis of current–voltage characteristics

**Fig. 8:**
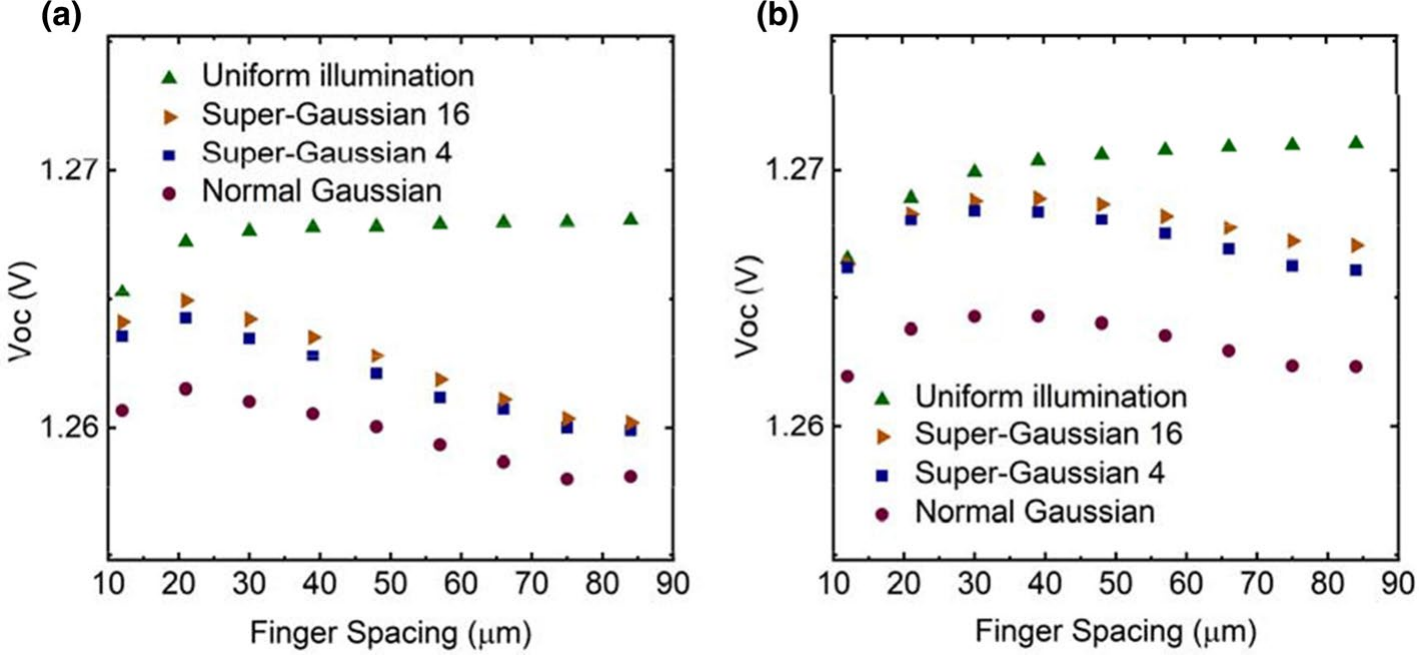
Open circuit voltage as function of finger spacing for **a** p–n and **b** n–p structure

**Fig. 9:**
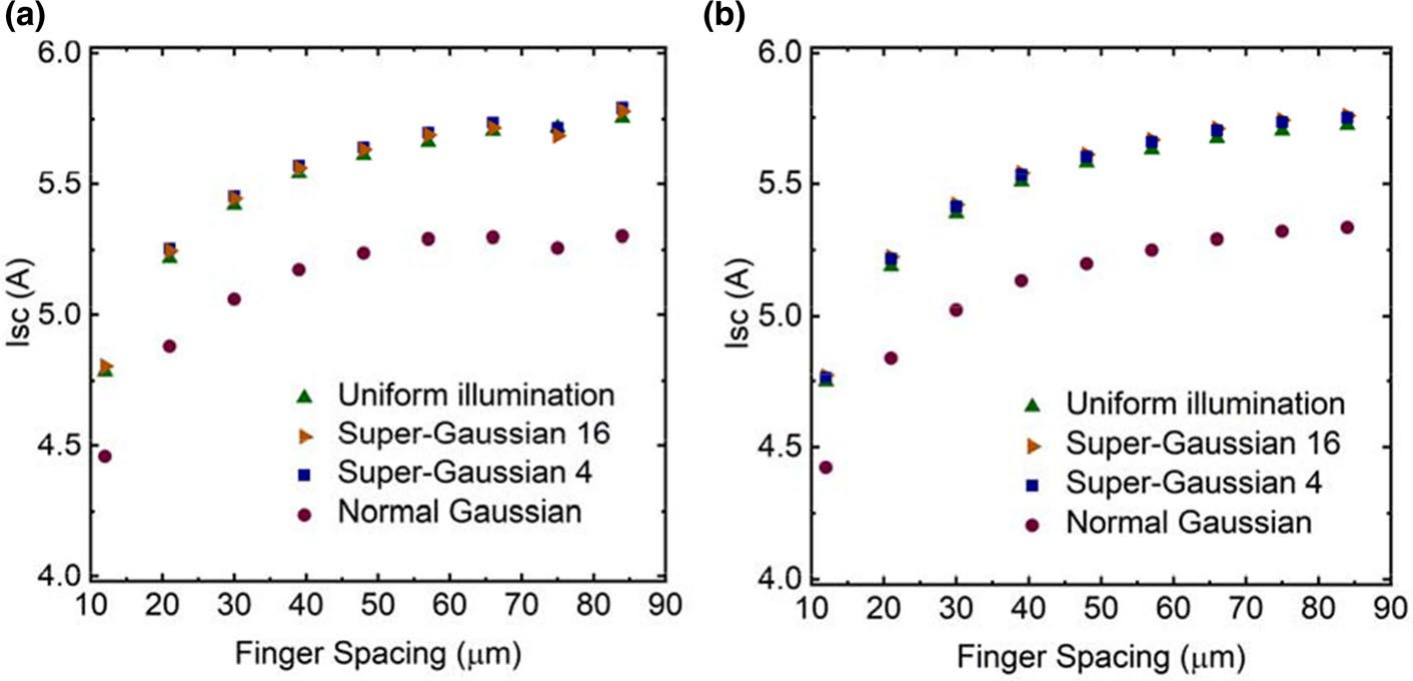
Short circuit current as function of finger spacing for **a** p–n and **b** n–p structure

**Fig. 10:**
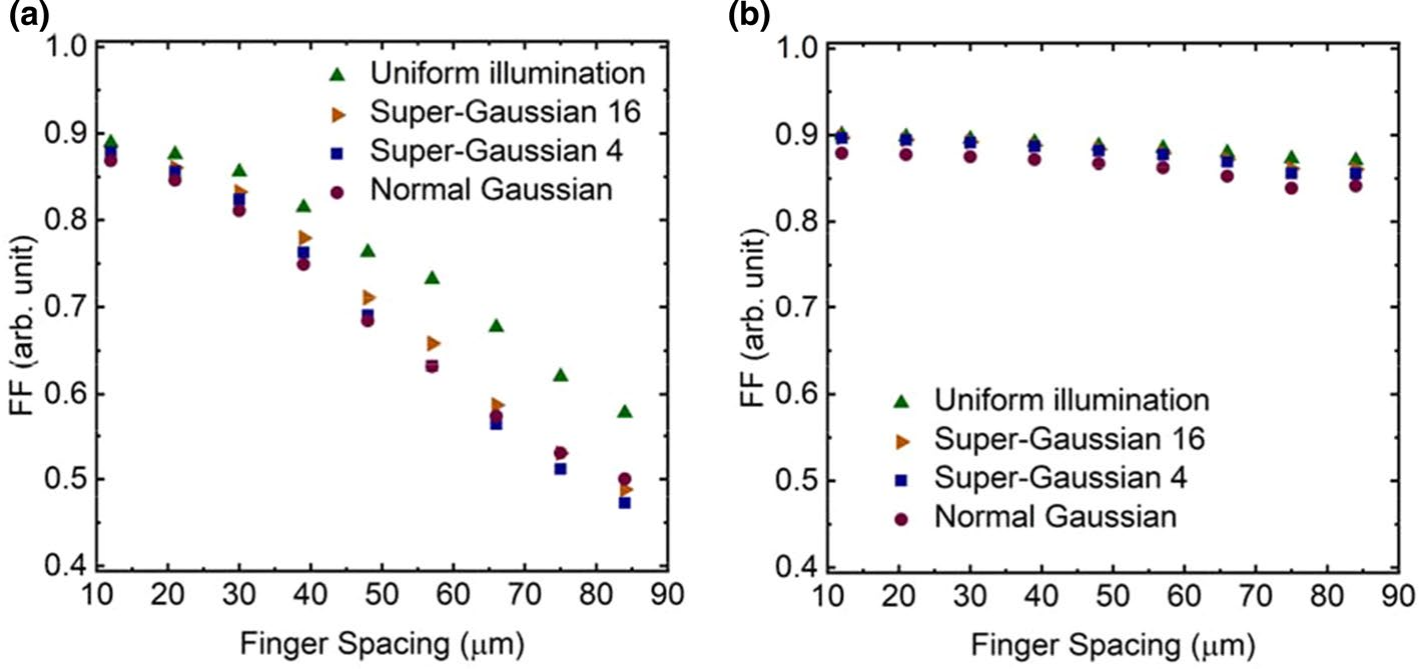
Fill factor as function of finger spacing for **a** p–n and **b** n–p structure

Figures 8, 9 and 10 shows the behavior of open circuit voltage (*V*_oc_), short circuit current (*I*_sc_) and fill factor (*FF*) of the PV cell as function of varying grid finger spacing at 300 K, respectively. It was found that for uniform illumination *V*_oc_ increases as spacing increases, but for other illuminations, it starts to decrease slightly approximately at the optimal spacing. *I*_sc_ increases as spacing increases, which is expected since the shading losses are reduced, and thus more light can enter the cell. The largest difference between the structures can be seen in *FF*. *FF* of the p–n structure decreases dramatically as the spacing increases, whereas for the n–p structure the effect is much milder. The maximum output power of PV cell is defined as

6 $$P_{out} = V_{oc} I_{sc} FF.$$

From this we can conclude that behavior of *FF* when finger spacing is varied is the main factor for observed differences in the *I*–*V* characteristics of the studied two structure variants. This difference is considered to arise from the fact that the p–n structure has higher resistive losses due to current transport in the p–type top layers compared to n–p structures, where n–type top layers spread the current. The p–type GaAs top layer has holes as majority carriers with mobility of ~ 150 ${\mathrm{cm}}^{2}/\mathrm{Vs}$ and the n–type top layer has electron mobility of ~ 2300 ${\mathrm{cm}}^{2}/\mathrm{Vs}$ at 300 K (Sotoodeh et al. 2000). These yield sheet resistance values for p- and n-emitter layers of ~ 222 $\Omega /\square$ and ~ 14 $\Omega /\square$, respectively. This difference is notable and thus dictates especially the device’s *FF* when finger spacing is widened.

The increase in resistive losses from non-uniformity of the illumination is evident in Fig. 10a, which reveals a reduction on the *FF* due to non-uniform illumination. The *FF* in the p–n structure with Normal Gaussian illumination is less sensitive for changes in the finger spacing compared to other non-uniform cases when spacing exceeds 50 µm. We assume this is the result from the smaller currents due to smaller total excitation. Another aspect for the losses of uneven illumination was seen form Fig. 8 where in addition to unbalanced current generation, the non-uniform illumination profile will cause a lateral unbalance in the voltage generated across the p–n or n–p junction, which induces further electrical loss. This electrical loss will lead to reduced *V*_oc_. Despite this fact, the resistive loss is still the dominant loss mechanism. For example, resistive losses causes ~ 4.2% power reduction for the n–p structure when the finger spacing is increased from 30 to 75 µm for Normal Gaussian illumination at 300 K, whereas the *V*_oc_ reduction due to non-uniform illumination profile causes only ~ 0.15% performance drop.

## Conclusions

A Hybrid Quasi-3D model was developed based on open-source Python-based library Solcore. It was used for optimization of the front contact grid for various photovoltaic converter structures. Characteristics of p–n and n–p structures were considered and compared. The n–p structure yielded considerably wider optimal grid finger spacing and higher output power than the p–n structure. The n–p structure also had a much higher tolerance for varying the finger spacing.

Increased operating temperature was found to slightly decrease the optimal spacing for both structures and decrease the tolerance for varying spacing in the n–p structure. Also, increased temperature leads to significantly reduced output power of converters which was expected.

From the results analysis we conclude that the illumination profile has much more impact on the output power of p–n structure when compared to n–p structure. Also, for both structure architectures, the optimal spacing was found to decrease as the illumination profile gets less uniform. Uneven illumination gives also a rise to a small reduction of *V*_oc_ of the cell.

Overall, the n–p structure has a great advantage over its p–n counterpart. This is mainly due to decreased sheet resistance in the emitter layer of the p–n structure due to larger majority carrier mobility, which manifests higher fill factors for the n–p structure. The enhanced lateral current flow in the n–p structure also gives the structure larger tolerance for the designing and manufacturing of the front contact grid and wider possibilities for different illuminations profiles.

The Hybrid Quasi-3D model can be seen as a valuable tool for extraction meaningful physical information from PV cell devices and it can be used for determination of the optimal grid profile for a given structure. It can be used for analysis of effects of illumination profiles and loss mechanisms in the devices. Although here the model was utilized for the optimization of two PV converter structures, it can also be applied to other PV devices as well, most notably, CPV solar cells.
